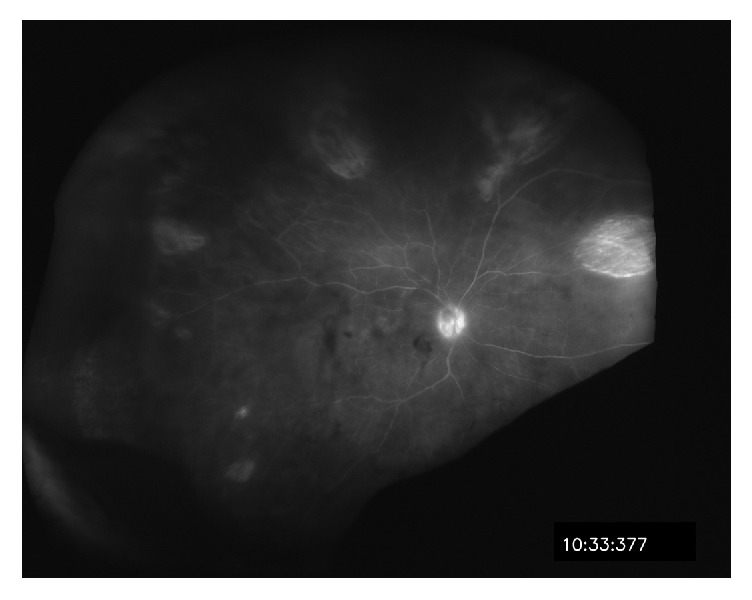# Concomitant Intravitreal Ganciclovir and Dexamethasone Therapy in the Management of Acute Retinal Necrosis in a Patient Previously Treated with Oral Famciclovir

**DOI:** 10.1155/2017/4613624

**Published:** 2017-08-08

**Authors:** Chirag V. Patel, Kamal Kishore

**Affiliations:** ^1^Capital Health Medical Center, Hopewell, NJ, USA; ^2^University of Illinois College of Medicine, Peoria, IL, USA

## Abstract

Several treatments have been reported for acute retinal necrosis (ARN). We report a case of treatment with intravitreal injection of ganciclovir and dexamethasone in addition to oral valacyclovir in a patient who was previously treated with oral famciclovir for ipsilateral herpes zoster ophthalmicus (HZO).

## 1. Introduction

Acute retinal necrosis (ARN) is a rare but well-recognized infectious uveitis resulting in rapidly progressive necrosis. The prognosis for vision recovery is guarded even with treatment [1] and very poor without treatment. Optimal treatment includes prompt control of both viral multiplication and inflammation for outpatient settings. Several treatments regimens have been reported since the initially reported successful outcomes with intravenous acyclovir [2] including intravitreal injection of antiviral with and without concurrent systemic antiviral and corticosteroids. Treatment with intravitreal injection of ganciclovir and dexamethasone in a patient already treated with oral antiviral medication has not previously been reported. In this case report, we describe a case indicating the utility of intravitreal injection of ganciclovir and dexamethasone in a patient previously treated with seven day course of oral famciclovir.

## 2. Case Presentation

**Figure 1:**
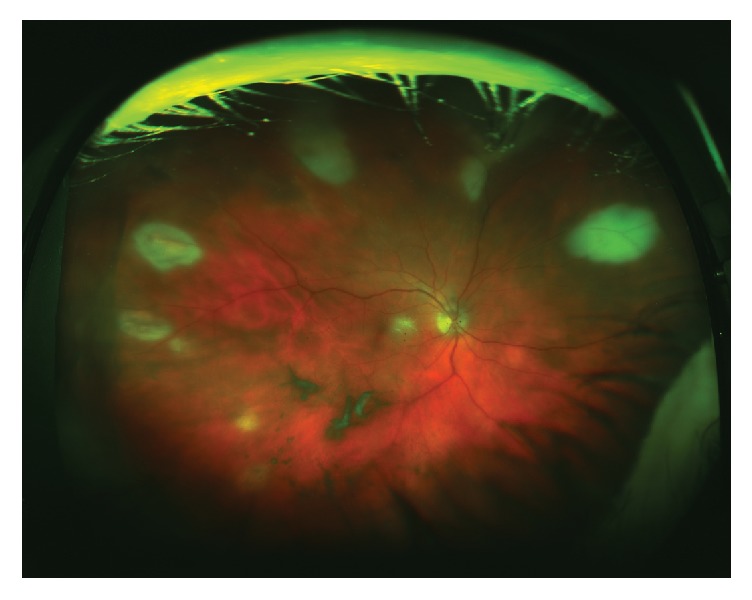


A 77-year-old male presented with unilateral panuveitis and peripheral multifocal retinitis (Figure 1) after previously being treated for ipsilateral herpes zoster ophthalmicus (HZO). Specifically, 5 weeks prior, he had been treated with famciclovir (Famvir) 500 mg every 8 hours for 7 days for his HZO. At the initial evaluation with his eye care provider, right eye visual acuity was 20/40-3. There was no ocular involvement associated with the external rash typical for HZO. A follow-up slit lamp examination 3 weeks later also did not show any ocular involvement. His past medical history was notable for rheumatoid arthritis, autoimmune hemolytic anemia, and recent onset jaundice.

Subsequently, he noted floaters and flashes. He also described discomfort around the right eye. Presenting right eye visual acuity was 20/80 with pin-holed improvement of 20/40-1. External examination was notable for healed vesicles in the ophthalmic division of the trigeminal nerve. Anterior segment inflammation consisted of granulomatous keratic precipitates (KP), mild cellular reaction, with an elevated IOP of 35 mm Hg. Posterior segment was notable for a moderate vitritis. There were well-defined 8 necrotic areas in the peripheral retina. There were segments of arteriolar sheathing within the areas of necrosis. The left eye was without inflammation. Examination of the posterior segment was notable for a posterior vitreous detachment and a horseshoe tear in the peripheral retina.

Acute retinal necrosis (ARN) was suspected and the patient was advised to have immediate aqueous and vitreous taps, followed by intravitreal ganciclovir 2 mg/0.1 ml injection and dexamethasone 0.4 mg/0.1 ml injection of the right eye. The patient was started on oral valacyclovir (Valtrex) 1 g/three times daily for fellow eye prophylactic treatment.

**Figure 2:**
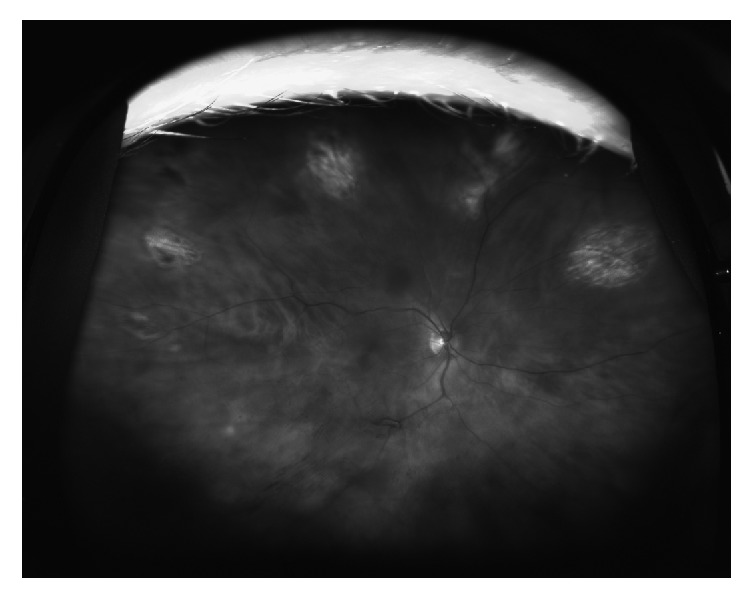


One week after treatment, right visual acuity was improved to 20/60 with pin-holed improvement to 20/25. The anterior chamber still showed moderate inflammation although most of the keratic precipitates had resolved. The location of the KP was mostly inferior. The lesions were noted to be partially regressed in size and density and no new lesions were detected in either eye (Figure 2). Qualitative polymerase chain reactions (PCR) for CMV, HSV-1, and HSV-2 were negative. VZV DNA was detected by PCR of the aqueous sample.

The patient was kept on oral valacyclovir 1 g/three times daily for 6 weeks total. Two months later, vision was measured to be 20/20-1. The previous areas of necrosis were replaced by sharply delineated atrophic areas with decreased vitreous debris (Figure 3). The patient remained free from recurrence two years after discontinuation of oral valacyclovir.

## 3. Discussion

ARN is a rare but well-recognized infectious uveitis resulting in rapidly progressive necrosis without prompt treatment. Initially it was identified as Kirisawa type uveitis [3] and subsequently as the currently recognized descriptor of ARN. The diagnostic criteria proposed by the American Uveitis Society in 1994 include single or multiple areas of distinct retinal necrosis, rapid progression without antiherpetic treatment, extension of necrosis in circumferential pattern, presence of occlusive vasculopathy with arteriolar involvement, and presence of anterior chamber and vitreous inflammation. Etiology of disease is reactivation of latent herpetic viral infection with herpes simplex virus (HSV) and varicella zoster virus (VZV) being the most common causes. Other members of the herpes virus family less often associated with ARN include cytomegalovirus and Epstein-Barr virus. While previously considered a disease affecting young and healthy individuals, ARN does affect both the immunocompetent and immunocompromised population. The immune status of the patient affects clinical course and outcome. The prognosis for vision recovery is guarded even with treatment [1] and very poor without treatment. Several treatment regimens have been proposed; however, given the low incidence, standard of care treatment is still based on the initially reported successful outcomes with intravenous acyclovir [2]. The schedule of 500 mg/m^2^ every 8 hours intravenously was modified to oral acyclovir after 10 to 14 days of intravenous treatment. More recently, with a higher bioavailability, oral agents such as famciclovir [4] and valaciclovir [5, 6] have been advocated as a replacement for initially administered intravenous and subsequently oral acyclovir. In addition, intravitreal antiviral treatments [7, 8] have been advocated as an adjunct to the required systemic treatment given the risk of fellow eye involvement [9]. Foscarnet and/or ganciclovir administered via intravitreal injection either as a one-time treatment or as induction followed by maintenance treatments have been reported. While there are cases of systemic treatments not being tolerated and therefore necessitating intravitreal antiviral treatment, primary intravitreal treatment may offer higher therapeutic levels of antiviral not achieved systemically due to the blood ocular barrier. Speculation exists as to whether oral antiviral agents alone are sufficient to treat all cases of intraocular herpetic disease or are useful to prevent fellow eye involvement [10]. Finally clinicians must consider, given the different members of the herpes virus family implicated in ARN, the disease may fail to respond to standard of care treatment.

The severe inflammatory response associated with ARN leads to vitritis and significant necrosis and later to development of proliferative vitreoretinopathy [11]. While treatment aimed at decreasing inflammation is initiated after antivirals are started [12], the inflammatory response may not be adequately treated using standard of care treatment [13]. Systemic corticosteroid and topical prednisolone are commonly used 48 hours after antiviral treatment. Since the inflammation can include an occlusive vasculitis and/or arteritis, anticoagulants and aspirin have been advocated although less commonly used. Culbertson's histopathology paper described inflammatory infiltration of the corneal endothelial surface, trabecular meshwork, iris, vitreous, optic nerve, retina, and choroid. Specifically in their case, both the optic nerve and retina were necrotic and the arteries in these affected structures were infiltrated with inflammatory cells. It was indeterminate if the arteritis resulted from contiguous viral infection or from deposition of immune complexes. The sudden and profound vision loss may be due to an arteritic optic neuropathy. By injecting ganciclovir and dexamethasone simultaneously, both the viral insult and immune-mediated reaction are addressed. The addition of dexamethasone allows for a safe and effective agent with a short half-life [14]. Our patient responded well to one injection each of ganciclovir and dexamethasone followed by oral valacyclovir although he presented with less than severe involvement of the peripheral retina and moderate vision loss. In a series reported by Meghpara et al. [15], patients with moderate disease (25–50% retinal involvement) did well with intravitreal injection of ganciclovir and/or foscarnet.

The incidence of ARN is rare and the clinical presentation is varied which make prospective studies difficult. Numerous case series report alternative treatment options that are comparable to standard of care in terms of efficacy. We believe that concomitant treatment with intravitreal ganciclovir and dexamethasone may address the direct viral burden as well as the secondary inflammation in a more localized manner. While a single report warrants further investigation prior to clinically significant conclusions, intravitreal dexamethasone used in conjunction with an intravitreal antiviral agent may be helpful in the management of ARN.

## Figures and Tables

**Figure 3:**